# Association between triglyceride glucose-body mass index and heart failure in subjects with diabetes mellitus or prediabetes mellitus: a cross-sectional study

**DOI:** 10.3389/fendo.2023.1294909

**Published:** 2023-11-03

**Authors:** Shuping Yang, Xiangxiang Shi, Wanlu Liu, Zhaokai Wang, Ruoshui Li, Xianzhi Xu, Chaofan Wang, Lei Li, Ruili Wang, Tongda Xu

**Affiliations:** ^1^ Department of General Practice, The Affiliated Hospital of Xuzhou Medical University, Xuzhou, Jiangsu, China; ^2^ Institute of Cardiovascular Disease Research, Xuzhou Medical University, Xuzhou, Jiangsu, China; ^3^ Department of Cardiology, The Affiliated Hospital of Xuzhou Medical University, Xuzhou, Jiangsu, China; ^4^ Department of Stomatology, The Affiliated Hospital of Xuzhou Medical University, Xuzhou, Jiangsu, China; ^5^ Department of Cardiology, Suining County Branch Hospital for the Affiliated Hospital of Xuzhou Medical University, Xuzhou, Jiangsu, China

**Keywords:** triglyceride glucose-body mass index, heart failure, diabetes mellitus, prediabetes mellitus, insulin resistance, NHANES

## Abstract

**Background:**

The triglyceride glucose-body mass index (TyG-BMI) is a surrogate indicator of insulin resistance. However, the association of TyG-BMI with heart failure (HF) in individuals with diabetes mellitus or prediabetes mellitus is unknown.

**Methods:**

This study included 7,472 participants aged 20–80 years old with prediabetes or diabetes from the National Health and Nutrition Examination Survey (2007–2018). The TyG-BMI was calculated as Ln [triglyceride (mg/dL) × fasting blood glucose (mg/dL)/2] × BMI, and individuals were categorized into tertiles based on TyG-BMI levels. The relationship of TyG-BMI with HF was analyzed using multiple logistic regression models. Subgroup analyses were stratified by gender, age, hypertension, and diabetes mellitus status.

**Results:**

This cross-sectional study had 7,472 participants (weighted n = 111,808,357), including 329 HF participants. Participants with a high TyG-BMI were prone to HF. The highest tertile group with a fully adjusted model was more likely to have HF compared to the lowest tertile group (odds ratio [OR], 2.645; 95% CI, 1.529–4.576). Restricted cubic spline analysis showed a significant dose-response relationship between TyG-BMI and HF (P < 0.001). In subgroup analyses, similar results were seen in terms of age (≥50 years old), gender, hypertension, and diabetes mellitus status.

**Conclusion:**

A high TyG-BMI is significantly associated with HF risk in participants with diabetes mellitus or prediabetes mellitus.

## Introduction

Heart failure (HF) is a systemic disease caused by multiple factors (1). The main features are damage to the structure and function of the heart and alterations in neurohormonal regulation (2). The mortality rate of hospitalized patients with HF is approximately 4% (3, 4), and the mortality rate of these patients within one month of discharge is 10% (5). One in five individuals older than 40 years will develop HF (6). This creates health hazards and adds pressure to the economy. Thus, early identification and intervention in individuals at high risk of developing HF are essential.

Many studies have shown that the incidence of HF is very high in the diabetes mellitus population (7), and diabetics with HF have a greater risk of death (8). Diabetic women have five times the risk of developing HF as non-diabetic women (9, 10). Prediabetes mellitus is the intermediate stage between normal blood glucose and diabetes mellitus, and it is characterized by impaired glucose metabolism (11). In a related study, prediabetes mellitus also increased the risk of HF, and prediabetic patients were more likely to develop ejection-preserved HF and have worse clinical prognosis (12). A high risk of HF in patients with diabetes mellitus or prediabetes mellitus may be related to insulin resistance (IR), but no study has examined the mechanism of action (13, 14).

The hyperinsulinemic euglycemia clamp technique is the gold standard for measuring IR (15). Because of economic cost and ethical issues, the application of the technique in clinical practice is difficult (15). Recently, the triglyceride glucose (TyG) index was found to be similarly sensitive and specific in predicting IR (16, 17), with triglyceride glucose-body mass index (TyG-BMI) predicting IR better than the TyG index. Therefore, our study explores the association of TyG-BMI with HF in patients with diabetes mellitus or prediabetes mellitus.

## Materials and methods

### Participants and study design

**Figure 1:**
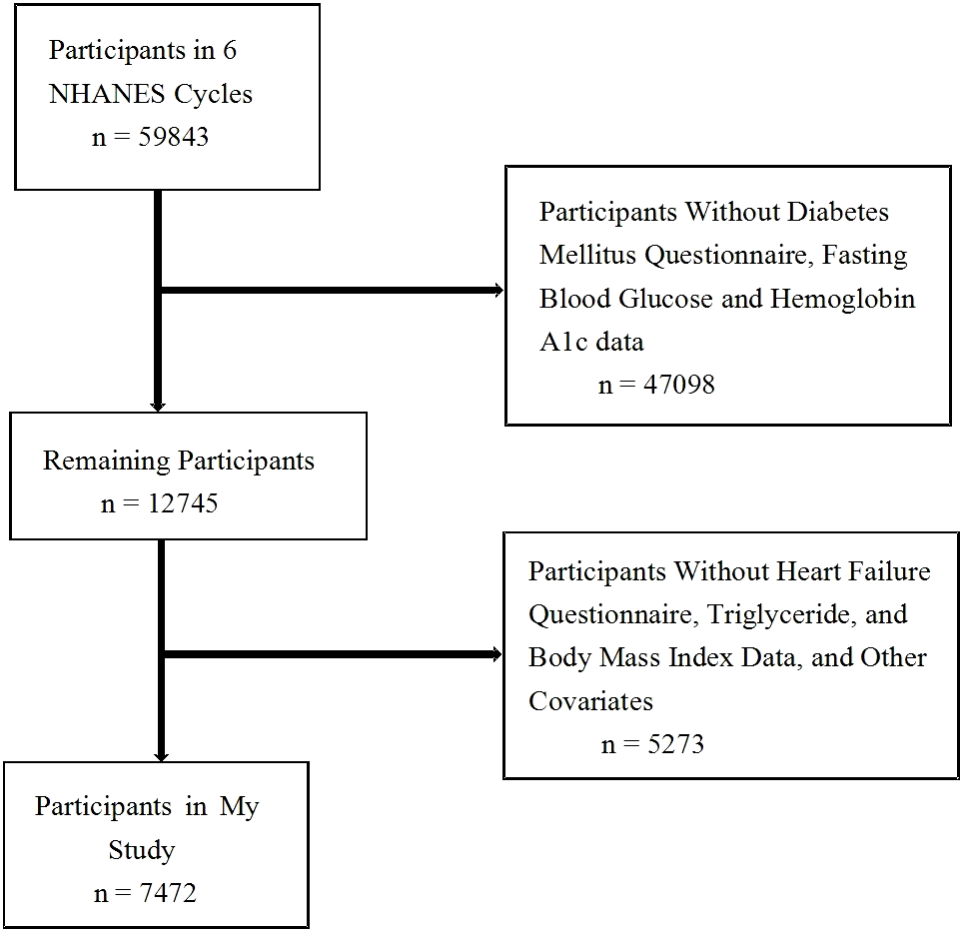
The flow chart of the systematic selection process.

The participants were selected from the National Health and Nutrition Examination Survey (NHANES) from 2007–2018. The National Center for Health Statistics, the heart of the Centers for Disease Control and Prevention, comprises NHANES, which is a study investigating the nutritional and health status of adults and children in the United States. A total of 12,745 individuals with fasting blood glucose (FBG) and hemoglobin A1c (HbA1c) data, as well as complete history of diabetes mellitus from 2007–2018 were included in the study. We excluded 5,273 participants for the following reasons: (1) participants without HF questionnaire data; (2) participants without BMI, TG, and/or FBG data; and (3) participants without other covariates. **Figure 1** shows the flow chart of the systematic selection process.

Diabetes mellitus and prediabetes mellitus were defined as follows: for diabetes mellitus, patients were informed by a doctor to have diabetes mellitus, or patients had FBG ≥126 mg/dl or HbA1c ≥6.5%. For prediabetes mellitus, patients had FBG 100–125 mg/dl or HbA1c 5.7%–6.4% (18). Finally, 7,472 participants were selected for analysis. The public database provided the data for this study, and written informed consent was obtained from the participants by NHANES.

### Assessment of HF

The NHANES questionnaire asks: “Has a doctor or other health professional ever told you that you have been diagnosed with HF?”. The participants who answered “yes” were considered to have HF.

### Data collection and measurements

Demographic data included age, gender, and race. Race included non-Hispanic White, Mexican American, other Hispanic, non-Hispanic Black, and other races. Questionnaire data included drinking status, smoking status, disease status (hypertension, coronary artery disease, and stroke), and medication status (ACE inhibitors, beta blocker, statin, and diuretics). Smoking was defined as smoking more than one hundred cigarettes in the past year (19). Drinking consumption was defined as having had 12 glasses of wine in the past year (20). The history of diseases was given to patients by a doctor. Measured data included BMI, systolic blood pressure (SBP), and diastolic blood pressure (DBP). Laboratory data collection was performed using fasting blood samples, including FBG, creatinine, HbA1c, estimated glomerular filtration rate (eGFR), low-density lipoprotein cholesterol (LDL-C), triglyceride (TG), high-density lipoprotein cholesterol (HDL-C), and total cholesterol (TC).

### Definition of index

The formulas for calculating the indexes were as follows.

TyG index = Ln [TG (mg/dL) × FBG (mg/dL)/2

BMI = weight (kg)/height (m)^2^

TyG-BMI = TyG index × BMI

### Statistical analyses

Qualitative variables were expressed as numbers (n) and percentages (%), and continuous variables were expressed as mean ± standard deviation. Participants were categorized into tertiles based on TyG-BMI. The chi-square test or Kruskal–Wallis H-test was used for analysis. Assessment of the nonlinear association between TyG-BMI and HF used restricted cubic spline curves. We used multiple logistic regression models to explore the correlation between TyG-BMI and HF. Model 1 was non-adjusted; model 2 was adjusted for age, gender, race, smoking status, and drinking consumption; model 3 was adjusted for model 2, LDL-C, eGFR, hypertension, coronary artery disease, ACE inhibitors, Beta blocker, stain, and diuretics.

In subgroups analyses, we stratified the analysis by hypertension (yes or no), gender (male or female) and age (< 50 or ≥ 50 years old), and diabetes mellitus status (diabetes mellitus or prediabetes mellitus) and assessed interactions by likelihood ratio tests. All analyses were performed using R version 3.6.1 (https://www.r-project.org/). Statistical tests were significant at P < 0.05.

## Results

### Baseline characteristics

In our study, our final analysis included 7,472 NHANES participants (weighted n = 111,808,357). The baseline characteristics of the patients according to the TyG-BMI tertiles: tertile 1 (TyG-BMI <232.72), tertile 2 (232.72 ≤ TyG-BMI < 286.33), and tertile 3 (TyG-BMI ≥286.33) are shown in **Table 1**. This study included 1,547 diabetes mellitus patients and 5,925 prediabetes patients, of which 329 (3.6%) had HF. The mean age of all individuals was 54.69 ± 16.10 years, with 46.2% males and 53.8% females. Participants of the T3 group showed higher total cholesterol, BMI, FBG, SBP, DBP, HbA1c, and TG than participants of the T1 group.

**Table 1:** Characteristics of participants in the study.

	Overall	Tertile 1	Tertile 2	Tertile 3	P value
N	7472	2491	2490	2491	
TyG-BMI	268.62(70.48)	201.31(23.16)	258.23(15.34)	346.32(56.10)	<0.001
Age (years)	54.69(16.10)	55.48(17.38)	55.48(15.86)	53.11(14.96)	<0.001
< 50	2761(41.90)	886(40.70)	881(40.50)	994(44.40)	
≥ 50	4711(58.10)	1605(59.30)	1609(59.50)	1497(55.60)	0.001
Male (n, %)	3962(53.80)	1416(56.10)	1409(56.50)	1137(49.00)	<0.001
Race (n, %)					<0.001
Mexican American	1214(9.30)	263(6.40)	471(10.80)	480(10.80)	
Other Hispanic	827(5.80)	238(5.20)	300(6.50)	289(5.90)	
Non-Hispanic White	2854(64.50)	943(64.30)	919(63.30)	992(65.70)	
Non-Hispanic Black	1554(11.60)	494(10.90)	483(10.90)	577(12.80)	
Other Race	1023(8.80)	553(13.20)	317(8.50)	153(4.80)	
Current smoker (n, %)	3448(47.10)	1121(46.80)	1158(47.40)	1169(47.00)	<0.001
Drinking consumption (n, %)	3806(75.40)	1266(76.40)	1314(77.10)	1226(72.90)	0.373
BMI (Kg/m2)	30.51(7.20)	24.04(2.65)	29.41(2.32)	38.09(6.38)	<0.001
HbA1c (%)	6.12(1.11)	5.81(0.72)	6.06(0.99)	6.5(1.39)	
FBG (mmol/L)	6.67(1.97)	6.08(1.10)	6.53(1.73)	7.39(2.56)	<0.001
SBP (mmHg)	127.66(17.37)	126.84(18.58)	127.71(16.66)	128.45(16.64)	0.015
DBP (mmHg)	70.67(12.68)	69.20(12.57)	70.74(11.93)	72.08(13.29)	<0.001
TC (mmol/L)	4.99(1.10)	4.90(1.06)	5.05(1.13)	5.01(1.09)	<0.001
TG (mmol/L)	1.61(1.52)	1.09(0.53)	1.60(0.93)	2.16(2.24)	<0.001
HDL (mmol/L)	1.36(0.39)	1.41(0.41)	1.35(0.38)	1.33(0.39)	<0.001
LDL (mmol/L)	2.83(0.95)	2.84(0.92)	2.83(0.96)	2.83(0.98)	0.992
eGFR	113.88(50.85)	88.88(32.57)	107.42(37.31)	145.30(58.53)	<0.001
Creatinine (umol/L)	80.80(38.56)	80.76(36.33)	81.7(34.90)	79.93(43.59)	0.364
Heart failure (n, %)	329(3.60)	76(2.50)	97(3.00)	156(5.10)	<0.001
Diabetes mellitus status (n, %)					<0.001
Prediabetes mellitus	5925(83.00)	2214(92.10)	2026(85.60)	1685(71.80)	
Diabetes mellitus	1547(17.00)	277(7.90)	464(14.40)	806(28.20)	
Hypertension (n, %)	3443(42.40)	877(30.40)	1169(42.80)	1397(53.50)	<0.001
Coronary artery disease	400(4.80)	136(4.80)	122(4.40)	142(5.30)	0.424
Stroke (n, %)	329(3.70)	101(3.60)	108(3.30)	120(4.20)	0.416
ACE inhibitors (n, %)	1901(27.00)	644(26.60)	595(27.40)	662(26.80)	0.080
Beta blocker (n, %)	1049(14.50)	372(14.90)	354(14.80)	323(13.80)	0.129
Statin (n, %)	2015(28.40)	677(27.70)	663(28.90)	675(28.60)	0.894
Diuretics (n, %)	1309(18.40)	435(18.70)	431(18.80)	443(17.70)	0.904

TyG-BMI, triglyceride glucose-body mass index; BMI, body mass index; HbA1c, hemoglobin A1c; SBP, systolic blood pressure; DBP, diastolic blood pressure; FPG, fasting plasma glucose; TC, total cholesterol; TG, triglyceride; HDL-C, high-density lipoprotein cholesterol; LDL-C, low-density lipoprotein cholesterol; eGFR, estimated glomerular filtration rate.

### The association of TyG-BMI with HF

**Figure 2:**
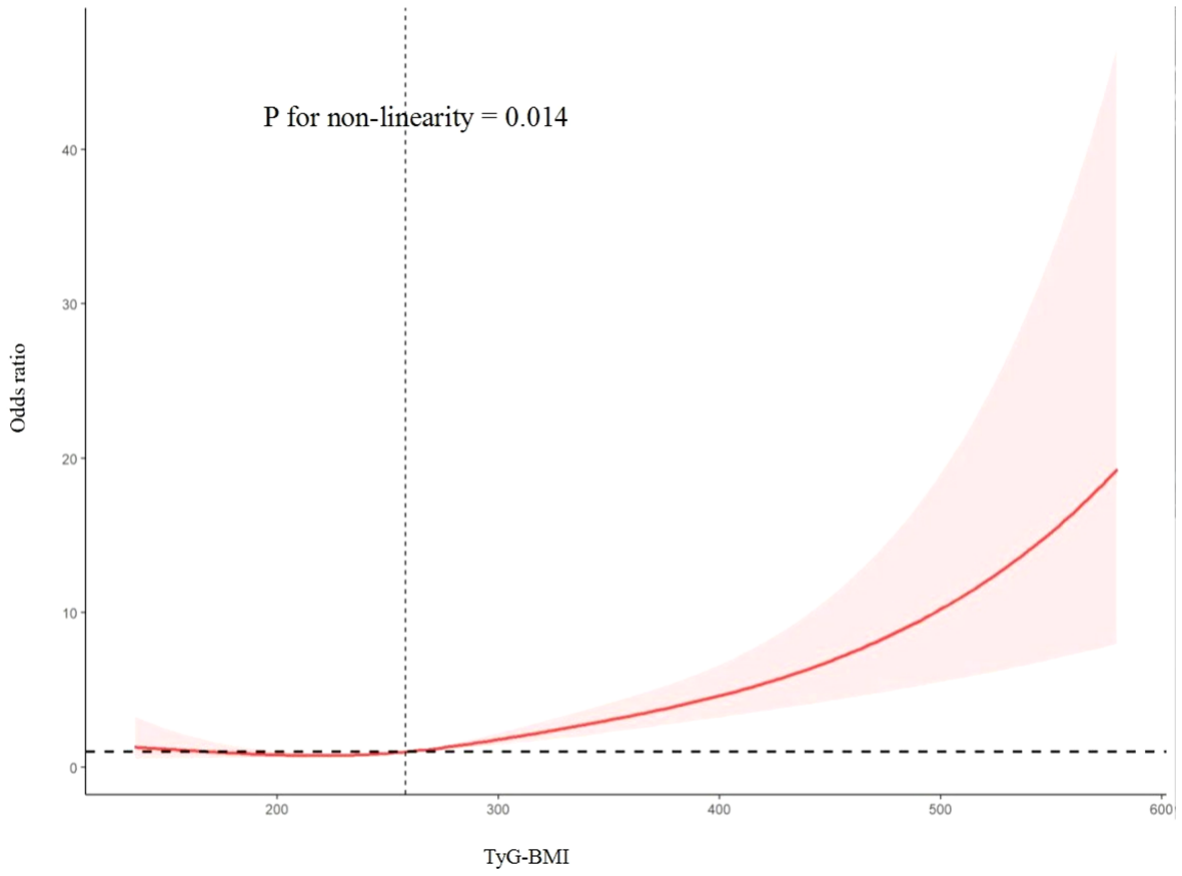
Restricted cubic spline curve for the association between TyG-BMI with heart faliure.

As shown in **Table 2**, logistic regression models showed the association of TyG-BMI with HF. When TyG-BMI was analyzed as a continuous variable, patients were more likely to have HF (OR, 1.009; 95% CI, 1.006–1.012). T3-group patients had higher odds of developing HF than T1-group patients in the fully adjusted model (odds ratio [OR], 2.645; 95% CI, 1.529–4.576). When TyG-BMI was converted to a categorical variable, the P for trend was also significant in unadjusted (P for trend, <0.001) or adjusted (P for trend, <0.001) models. In **Figure 2**, using a multiple logistic regression model with cubic spline functions, we observed a dose-response relationship of TyG-BMI with HF (non-linear P < 0.05), and a higher TyG-BMI (>258.26) was associated with an increased HF risk.

**Table 2:** Relationship between TyG-BMI and heart failure.

Exposure	Model 1	Model 2	Model 3
TyG-BMI	1.005(1.003,1.007)<0.001	1.007(1.005,1.010)<0.001	1.009(1.006,1.012)<0.001
TyG-BMI tertile			
T1	1.0	1.0	1.0
T2	1.218(0.728,2.036)0.448	1.286(0.759,2.178)0.346	1.247(0.743,2.092)0.399
T3	2.121(1.339,3.358)0.002	2.712(1.625,4.525)<0.001	2.645(1.529,4.576)<0.001
P for trend	<0.001	<0.001	<0.001

Data are presented as odds ratios, 95% confidence intervals, and P-value.

Model 1 was non-adjusted; model 2 was adjusted for gender, age, race, smoking status, and drinking consumption; model 3 was adjusted for model 2, LDL-C, eGFR, hypertension, coronary artery disease, ACE inhibitors, Beta blocker, stain, and diuretics.

### Subgroup analyses

**Figure 3:**
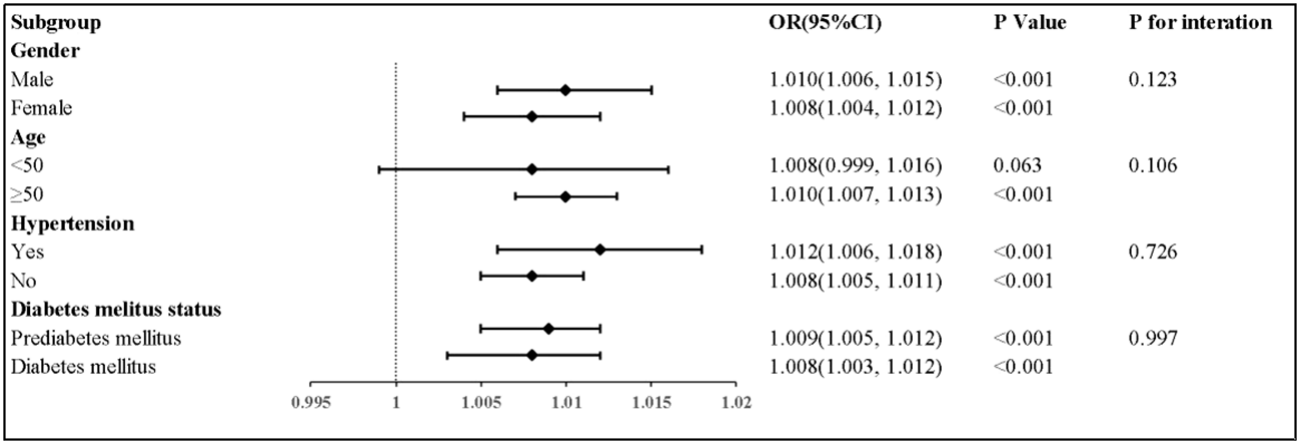
Subgroup analyses for the association between TyG-BMI with heart faliure. Adjusted for gender, age, race, smoking status, and drinking consumption, LDL-C, eGFR, hypertension, coronary artery disease, ACE inhibitors, Beta blocker, stain, and diuretics.

The results of the subgroup analyses are shown in **Table 3** and **Figure 3**. For participants aged ≥50 years, TyG-BMI was significantly associated with a higher HF prevalence. Subgroup analyses of gender and hypertension showed significant associations between TyG-BMI and HF prevalence. Finally, a higher TyG-BMI associated with a greater HF prevalence in diabetes mellitus (OR, 1.008; 95% CI, 1.003–1.012) and prediabetes mellitus (OR, 1.009; 95% CI, 1.005–1.012) patients. No obvious interactions between TyG-BMI and HF in all subgroup analyses (P for interaction, >0.05) were noted.

**Table 3:** Subgroup analyses for the association between TyG-BMI index and heart faliure.

Subgroup	OR(95%CI)	P Value	P for interation
Gender			
Male	1.010(1.006, 1.015)	<0.001	0.123
Female	1.008(1.004, 1.012)	<0.001	
Age			
<50	1.008(0.999, 1.016)	0.063	0.106
≥50	1.010(1.007, 1.013)	<0.001	
Hypertension			
Yes	1.012(1.006, 1.018)	<0.001	0.726
No	1.008(1.005, 1.011)	<0.001	
Diabetes mellitus status			
Prediabetes mellitus	1.009(1.005, 1.012)	<0.001	0.997
Diabetes mellitus	1.008(1.003, 1.012)	<0.001	

Adjusted for sex, gender, race, smoking status, and drinking consumption, LDL-C, eGFR, hypertension, coronary artery disease, ACE inhibitors, Beta blocker, stain, and diuretics.

## Discussion

This cross-sectional study explored the association of TyG-BMI with HF in diabetic and prediabetic populations from NHANES 2007–2018. Our study confirmed that diabetic or prediabetic participants with high TyG-BMI were more likely to have HF. When the index was used as a categorical or continuous variable, TyG-BMI was significantly associated with increased HF risk. Restricted cubic spline analysis indicated that high TyG-BMI had a dose-response relationship with HF development. The risk is higher when the index is greater than 258.26.

Previous studies have confirmed that the TyG index is a surrogate marker for IR (16, 21). BMI is the simplest measure to assess body fat and metabolism (22). TyG-BMI is the multiplication of the TyG index and BMI. Er et al. indicated that TyG-BMI was better than the TyG index, BMI, conventional lipid levels, visceral obesity index, lipid accumulation products, and adipokine and lipid ratios in predicting IR (23). Liu et al. reported a significantly increased incidence of atherosclerotic cardiovascular disease in Taiwanese adults with increasing TyG-BMI (24). In another study, a linear relationship of TyG-BMI with ischemic stroke in the general population was demonstrated (25). A cross-sectional study has indicated that individuals with a high TyG-BMI are more likely to have prehypertension (26), whereas a prospective study using data from Hong Kong and Taiwan found that individuals with a high TyG index were more likely to have HF (27). Xu et al. also reported that the TyG index was significantly associated with the prevalence of HF (28). The differences between our study and these two studies may be related to the fact that TyG-BMI predicted IR better than the TyG index, and previous cohorts were from the general population. Our study included diabetic and prediabetic patients.

In our subgroup analyses, fully-adjusted models showed a positive relationship between TyG-BMI and HF in individuals aged ≥50 years but not in those aged <50 years, which is probably due to the fact that fewer individuals aged <50 years developed HF than those aged ≥50 years in our study. Meanwhile, our study indicated that males with elevated TyG-BMI had a higher risk of developing HF than females. Although females are less likely to develop IR than males (29), HF due to high BMI and diabetes mellitus is more common in females (30), and diabetic females are more likely to develop coronary heart disease than males (31, 32). In summary, our findings are inconsistent with previous studies.

The TyG-BMI was associated with increased HF risk in diabetes mellitus status subgroup analysis, and the P for interaction was not significant, indicating TyG-BMI can be used to reflect HF risk of prediabetic and diabetic populations. In prediabetic and diabetic populations, HF occurrence was more likely to be induced by IR. Currently, the association between IR and HF can be explained by several mechanisms. Firstly, IR leads to cardiac pressure overload or ischemia susceptibility, which is possibly caused by impaired glucose utilization and subsequent increased free fatty acid oxidation (33, 34). Secondly, IR leads to adverse activation of the sympathetic nervous system, with chronic hyperinsulinemia increasing angiotensinogen release from adipose tissues and angiotensin II receptor expression, ultimately leading to aberrant cardiac remodeling and cardiac dysfunction (35). However, the mechanism of action between IR and HF requires further study.

The current study has several limitations. Firstly, our study is a cross-sectional observational study. Further prospective cohort studies, which include a control group, are likely to provide more real-world evidence in the future. Secondly, the diagnosis of HF may have been under- or over-reported by individuals in the questionnaire. Thirdly, the impact of the oral glucose tolerance test results on the diagnosis of prediabetes or diabetes was not considered.

## Conclusions

In conclusion, a high TyG-BMI was significantly associated with an increased HF risk in patients with diabetes mellitus or prediabetes mellitus. Treatments aimed at reducing TyG-BMI for HF prevention are urgently required.

## Data availability statement

The datasets presented in this study can be found in online repositories. The names of the repository/repositories and accession number(s) can be found below: https://www.cdc.gov/nchs/nhanes.

## Ethics statement

The present study received approval from the National Center for Health Statistics and database obtained consent from the participants. The studies were conducted in accordance with the local legislation and institutional requirements. The participants provided their written informed consent to participate in this study.

## Author contributions

SY: Data curation, Methodology, Writing – original draft. XS: Data curation, Methodology, Writing – original draft. WL: Data curation, Methodology, Writing – review & editing. ZW: Data curation, Methodology, Writing – review & editing. RL: Data curation, Writing – original draft. XX: Data curation, Writing – original draft. CW: Data curation, Writing – original draft. LL: Writing – review & editing. RW: Writing – review & editing. TX: Writing – review & editing.
